# Knee Cartilage Injuries in Football Players: Clinical Outcomes and Return to Sport After Surgical Treatment: A Systematic Review of the Literature

**DOI:** 10.1177/19476035231224951

**Published:** 2024-04-23

**Authors:** Luca Andriolo, Theodorakys Marín Fermín, Giulia Marcella Maryse Chiari Gaggia, Andreas Serner, Elizaveta Kon, Emmanuel Papakostas, Andrew Massey, Peter Verdonk, Giuseppe Filardo

**Affiliations:** 1 Clinica Ortopedica e Traumatologica 2, IRCCS Istituto Ortopedico Rizzoli, Bologna, Italy; 2 Centro Profesional Las Mercedes, Caracas, Venezuela; 3 IRCCS Humanitas Research Hospital, Milan, Italy; 4 FIFA Medical, Fédération Internationale de Football Association, Zurich, Switzerland; 5 Department of Biomedical Sciences, Humanitas University, Milan, Italy; 6 Aspetar Orthopaedic & Sports Medicine Hospital, Doha, Qatar; 7 ORTHOCA, Antwerp, Belgium; 8 Department of Orthopaedic Surgery, Antwerp University Hospital, Edegem, Belgium; 9 Service of Orthopaedics and Traumatology, Department of Surgery, Ente Ospedaliero Cantonale, Lugano, Switzerland; 10 Applied and Translational Research (ATR) Center, IRCCS Istituto Ortopedico Rizzoli, Bologna, Italy; 11 Faculty of Biomedical Sciences, Università della Svizzera Italiana, Lugano, Switzerland

**Keywords:** football, soccer, return to sport, cartilage, osteochondral

## Abstract

**Objective:**

To systematically review the literature and analyze clinical outcomes and return-to-sport after surgical management of cartilage injuries in football players.

**Design:**

A systematic literature review was performed in August 2023 on PubMed, WebOfScience, and Cochrane Library to collect studies on surgical strategies for cartilage lesions in football players. Methodological quality and risk of bias were assessed with the modified Coleman Methodology score and RoB2 and RoBANS2 tools.

**Results:**

Fifteen studies on 409 football players (86% men, 14% women) were included: nine prospective and two retrospective case series, one randomized controlled trial, one prospective comparative study, one case report, and one survey. Bone marrow stimulation (BMS) techniques were the most documented. The lesion size influenced the treatment choice: debridement was used for small lesions (1.1 cm^2^), BMS, osteochondral autograft transplantation (OAT), matrix-assisted autologous chondrocytes transplantation (MACT), and scaffold-augmented BMS for small/mid-size lesions (2.2-3.0 cm^2^), and autologous chondrocytes implantation (ACI) for larger lesions (5.8 cm^2^). The surgical options yielded different results in terms of clinical outcome and return-to-sport, with fastest recovery for debridement and scaffold-augmented BMS. The current evidence is limited with large methodological quality variation (modified Coleman Methodology score 43.5/100) and a high risk of bias.

**Conclusions:**

Decision-making in cartilage injuries seems to privilege early return-to-sport, making debridement and microfractures the most used techniques. The lesion size influences the treatment choice. However, the current evidence is limited. Further studies are needed to confirm these findings and establish a case-based approach to treat cartilage injuries in football players based on the specific patient and lesion characteristics and the treatments’ potential in terms of both return-to-sport and long-term results.

**Level of evidence:**

Systematic review, level IV.

## Introduction

Football (also known as soccer) is one of the most practiced sports in the world, involving more than 200 million players and 211 national associations.^1,2^ The overall incidence rate of injury in football is estimated to be of about 10 to 35 per 1,000 playing hours,^2,3^ and the knee is the most involved joint.^4^ Knee injuries may involve any joint structures, as cruciate or collateral ligaments, menisci, and cartilage. In particular, a cartilage lesion was reported in up to 60% of knee arthroscopic evaluation of high-impact sport athletes.^5^ A combination of anterior cruciate ligament (ACL) and cartilage lesions leads to a 5-fold increased risk for subsequent osteoarthritis compared to patients with only ACL lesions,^6^ and overall, 32% to 49% of former professional football players are diagnosed with osteoarthritis, predominantly in the knee and hip.^7^ Cartilage lesions and osteoarthritis can cause significant time-loss of both training and matches and may even result in sport retirement, as reported in up to 24% of the players who retire from sport due to injury.^8^

These outcomes underline the importance of properly and timely addressing knee joint lesions, particularly cartilage lesions, with the aim to allow a return to previous sport level and to reduce the risk of early degenerative changes in the knee.^9^ Multiple procedures are available for the management of cartilage lesions. These include bone marrow stimulation (BMS) techniques, osteochondral autograft transplantation (OAT) or mosaicplasty, fresh osteochondral allograft transplantation, autologous chondrocytes implantation (ACI) or matrix-assisted autologous chondrocytes transplantation (MACT), scaffold-augmented BMS, and cell-free chondral and osteochondral scaffolds. Good to excellent postoperative results in terms of subjective symptoms and functional improvement have been documented in the general population following the management of these lesions.^10-14^ However, less is known about the management of cartilage defects and the potential outcomes in terms of clinical results, return-to-sport rate and time, as well as complications and failures, in high-level athletes, specifically football players.

The aim of this study was to systematically review the literature and to summarize and analyze the existing data on clinical outcomes and return-to-sport after the different surgical strategies for the management of cartilage lesions in football players.

## Materials and Methods

A review of the current literature was performed using three databases (Pubmed, Web Of Sciences, and Cochrane Collaboration library) on August 7, 2023, according to the review protocol registered on Protocols.io (Protocol Integer ID: 81449). The search was conducted with no time nor language limitation, and without any filter, using the following string: (football OR soccer OR sport) AND (knee) AND (cartilage OR chondral OR osteochondral OR subchondral) AND (treatment OR surgery OR procedure OR technique). The PRISMA (Preferred Reporting Items for systematic Reviews and Meta-Analysis) guidelines were used (Supplementary material).^15^

The first screening was performed separately by two independent observers (TMF and GMMCG), and articles were sorted by title and abstract. The inclusion criteria for selection were as follows: (1) Participants included football players, injury description included knee cartilage injuries, and management included a surgical procedure, (2) study designs included randomized controlled trials (RCTs), prospective studies, retrospective studies, and case reports. Exclusion criteria were articles not containing specific data related to football players.

For the second screening, the full texts were retrieved and screened independently by both observers to identify relevant studies and exclude those who did not fit the criteria. Once a definite list of studies to be included was established, the relevant data were extracted independently by both observers to be analyzed for the purpose of this study. The information retrieved included: year of publication, type of study, number of football players included, age, sex, body mass index (BMI), level of play, mean follow-up, lesion size, lesion location, lesion etiology, lesion classification, surgical procedure performed, number and type of combined procedures, number and type of previous procedures, rehabilitation protocol, failure definition and rate, re-intervention rate, complications, results, return-to-sports (to any level and to the same level), and time to return-to-sport. For the assessment of the methodological quality of the analyzed studies, the modified Coleman Methodology Score (CMS) by Kon *et al.*^16^ was applied, and for the assessment of the risk of bias, the RoB 2 and RoBANS 2 tools were applied.^17,18^ Results were evaluated and integrated, and any discrepancies were discussed and resolved with a third author (LA).

## Results

**Figure 1.**
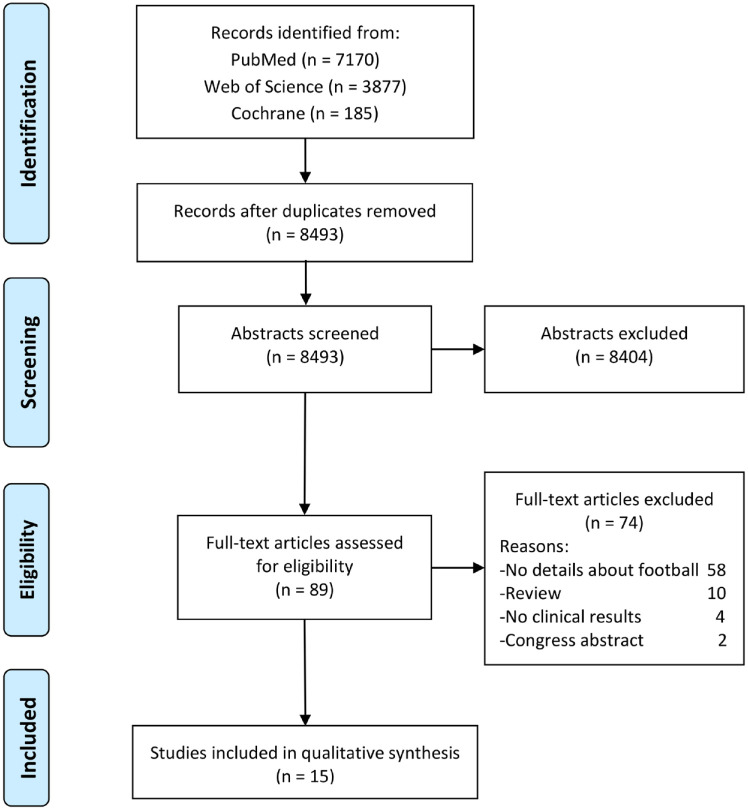
PRISMA flow-chart of the systematic literature review.

The search identified a total of 8,493 papers after the elimination of duplicates (Figure 1).

The articles were screened according to the inclusion and exclusion criteria, leaving a total of 89 full-text articles assessed for eligibility. Finally, 15 studies^19-33^ met the predefined eligibility criteria and were included in the review: nine prospective case series,^20,22,25,27-31,33^ two retrospective case series,^23,32^ one RCT,^21^ one prospective comparative study,^24^ one case report,^19^ and one survey.^26^ Details about the included studies are reported in Table 1 and Table 2.

**Table 1.** Study Characteristics.

Author, Journal, Year	Type of Study	N° Football Players (/N° Total Pts)	Age Sex	Football Level	Follow-up	Lesion Size (cm^2^)	Lesion Location	Lesion Etiology	Cartilage Procedures (details)	Combined Procedures	Rehabilitation Protocol
Beyzadeoglu *et al.*^19^	Case report	**1**	**27 y** **1 M**	**Professional**	**24 m**	**9**	**LFC**	**Degen**	**MACI** **(+ fibrin glue, open procedure)**	/	**Non-WB: w. Partial WB at w. Gradual progress to full WB at 12 w. At m low velocity running. PT and fitness program for 9 m**
Gobbi et al.^20^	Prospective study	**8**/53	**27.4 y** **7 M, 1 F**	**Competitive, recreational**	72 m	**5**	**7 MFC, 1 LFC**	**Trauma**	**MFX**	11 ACL, 3 lateral releases, and 3 meniscal suture	**WB surface: Crutch assisted touchdown WB 4 to 6 w. Full WB 8 w.** PF joint lesions: Brace 8 w, partial WB 1 w. Hydrotherapy and bicycling at 2 w. Full WB 8 w. Active functional training at 9 w. Sports training at 4 m, and then RTS.
Gudas *et al.*^21^	RCT	**13**/57	24.3 y 35 M, 22 F	23 highly competitive, 34 well trained	37.1 m	OAT: 2.8 MFX: 2.8	84% MFC, 16% LFC	56% posttrauma, 44% OCD	28 OAT vs 29 MFx (All-arthroscopic procedures)	/	Partial WB (20 kgs) after 4 w. Progression to full weight by 8 w. Depending on the clinical examination, patients were allowed to gradually return-to-sports in 4–6 m postoperatively.
Kacprzak *et al.*^22^	Prospective study	**36**/49	30 y 34 M, 15 F	**Professional**	19.8 m	3.0	20 MFC, 29 LFC	32 trauma, 11 OCD, 6 nontrauma	Scaffold-augmented BMS (arthroscopic MFx + Hyalofast scaffold implantation)	19 men	**No brace, immediate full load, crutch for 7 d. No ROM limitation. Resistance training, eccentric–concentric exercises, proprioception exercises. 3 w preliminary training sessions. RTS in 2.5-3 m**
Keszég *et al.*^23^	Retrospective study	**55**	**29.2 y** **40 M, 15 F**	**7 professional players, 23 competitive, 25 recreational**	**17.5 y**	**2.0**	**37 MFC, 13 LFC, 6 pat, 1 tr**	/	**OAT** **(19 arthroscopic, 36 arthrotomy)**	**7 HTO, 16 ACL, 2 lateral releases**	/
Kon *et al.*^24^	Prospective comparative study	**41**	**MFX: 26.5 y, MACT: 23.7 y** **41 M**	**Professional or semiprofessional**	**MFX: 89 m** **MACT: 94 m**	**MFX: 1.9 MACT: 2.1**	**MFX: 60% MFC, 20% LFC, 15% tr, 5% MFC + LFC.** **MACT: 62% MFC, 19% LFC, 9% tr, 5% MFC + tr, 5% LFC + tr**	/	**20 MFX vs 21MACT** **(arthroscopic Hyalograft C)**	**MFX: 4 ACL, 1 MCL, 1 HTO, 1 loose body removal, 1 calcification removal, 3 men, 2 patellar deb.** **MACT: 10 ACL, 10 men, 2 meniscal suture, 1 loose body removal**	**No WB for 4 w. Early mobilisation. Partial WB by w 3-4 with crutches, complete WB by 6-8 w. Gait training, return to normal running mode, and then sports specific training.**
Levy *et al.*^25^	Prospective study	**15**	**/** **9 M, 6 F**	**Competitive, professional**	**12 m**	**1.1**	**9 MFC, 5 LFC, 5 pat, 3 tr, 1 TP**	**40% trauma, 60% degen**	**Arthroscopic debridement**	**None**	**Cryotherapy and aggressive PT. Full WB with return of quadriceps strength at circa 1w. Then lower extremity strengthening. Progression to straight-line jogging and sprinting, and finally cutting and pivots. RTS when pain free.**
Marom *et al.*^26^	Multicenter Descriptive survey	**4,526**	**27 y** **3,395 M, 1,131 F**	**Professional: 10%**	/	**MFC: 2** **LFC: 2** **Pat: 2** **Tr: 2** **MTP: 1.8** **LTP: 1.5**	**30% MFC** **26% LFC** **16% pat** **15% tr** **6% MTP** **5% LTP**	**Trauma: 40%**, **Congenital: 10%**, **Comb T+C: 2%**, **Overload: 15%**, **OA/degen: 10%**	**Nonoperative 50%: PT, IA steroids, IA-HA, IA-PRP, IA-ASC, IA BMAC.** **Operative 50%: debridement, ORIF, BMS, OAT, allograft, ACI/MACT, Coronal osteotomy, TTO**	/	/
Mithoefer *et al.*^27^	Retrospective study	**21**	**27 y** **21 M**	**Professional**	**1-13 y**	/	**3 MFC, 7 LFC, 1 LTP, 1 pat, 9 multi**	/	**MFX**	**2 ACL**	**WB surface: 20%-30% WB with crutch for 8 w. Full WB and active ROM at 8 w. No cutting, turning, or jumping for 4-9 m.** **PF lesions: Brace locked at 0°. Full WB at 2 w. Walking with a brace at 8 w. Active walking at 12 w**.
Mithöfer *et al.*^28^	Prospective study	**4**/20	15.9 y 15 M, 5 F	1 competitive, 3 recreational	47 m	**7.1**	14 MFC, 6 LFC, 4 tr, 2 TP, 1 pat	Trauma 39%, OCD 61%	ACI with preiosteal flap (open procedure, “sandwich technique” in 2 pts)	1 ACL, 2 meniscal repair, 1 TTO	CPM for 2 w. Non-WB for 6-8 w, gradual progression to full WB by 10-12 w. Return to regular daily activities by 4 m. Low-impact by 6 m, progression to running at 9 m. Demanding/high-impact/pivoting sports avoided for 12 m.
Mithöfer *et al.*^29^	Prospective study	**45**	**26 y** **32 M, 13 F**	**33 recreational, 12 professional**	**41 m**	**5.7**	**48% MFC, 23% LFC, 13% Tr, 11%Pat, 5% PT**	**/**	**ACI with preiosteal flap** **(open procedure, “sandwich technique” if lesion deeper than 1 cm)**	**8 ACL, 4 TTO, 2 HTO, 2 meniscal repair, 2 HTO+ACL, 1 HTO+TTO**	/
Pánics *et al.*^30^	Prospective study	**61**	**25.3 y** **55 M, 6 F**	**Elite, competitive**	**9.6 y**	**2.4**	**38 MFC, 15 LFC, 3 LTP, 4 pat, 1 tr**	**/**	**Mosaicplasty**	**69%: HTO, ACL, men, PF realignement.**	/
Zaffagnini *et al.*^31^	Prospective study	**26**/31	22.6 y **26 M**	Professional, competitive	**10 y**	2.1	18 MFC, 8 LFC, 2 tr, 3 multi	48% trauma, 32% OCD, 20% microtrauma/degen	**MACT** **(arthroscopic Hyalograft C)**	/	Full WB at 6–8 w. Then functional training. Rehabilitation tailored according to combined surgeries and specific patients and sports requirements.
Zarur Mina *et al.*^32^	Retrospective study	**34**	**24.6 y** **34 M**	**Professional**	**30 m**	/	**34 Femoral condyles**	**Trauma**	**MFX**	**12 men**	**No/partial WB: 6-8 w dependent on size and location of defect. Specific physical therapy and rehabilitation program on day 4 or 5. RTS when strength was >80% compared to the healthy limb.**
Zmerly *et al.*^33^	Prospective study	**49**	NA	/	NA	/	**37 MFC, 9 LFC, 1 pat, 2 tr**	/	**37 perforations, 12 MFX**	/	/

Entries in boldface refer exclusively to data about football players.

pts = patients; M: male; F = female; y = year; m = months; w = week; RCT = randomized controlled trial; MFC = medial femoral condyle; LFC = lateral femoral condyle; pat = patella; tr = trochlea; TP = tibial plateau; MTP = medial tibial plateau; LTP = lateral tibial plateau; OCD = osteochondritis dissecans; degen = degenerative; OA = osteoarthritis; IA = intra-articular; HA = hyaluronic acid; PRP = platelet-rich-plasma; ORIF = open reduction internal fixation; men = meniscectomy; ACL = anterior cruciate ligament; MCL = medial collateral ligament; HTO = high tibial osteotomy; TTO = tibial tuberosity osteotomy; PF = patellofemoral; deb = debridement; RTS = return-to-sport; ROM = range of motion; PT = physiotherapy; WB = weight-bearing; MFX = microfractures; BMS = bone marrow stimulation; OAT = osteochondral autograft transplantation; ACI = autologous chondrocyte implantation; MACT = matrix-assisted autologous chondrocyte transplantation ; MACI = Matrix-assisted Autologous Chondrocyte Implantation; NA = not assessed.

**Table 2.** Study Results.

Author, Journal, Year	Type of Study	N° Football Players (/N° Total pts)	Cartilage Procedures (Details)	Outcome Meaures (fup)	Failures	Complications	Results	Return-to-Sport To Same Level %	Return-to-Sport to Any Level %	Time to Return	Coleman Methodology Score
Beyzadeoglu *et al.*^19^	Case report	**1**	**MACI** **(+ fibrin glue, open procedure)**	**Tegner-Lysholm and Brittberg-peterson**	**0**	**0**	**Improvement in clinical scores, good filling, and signal at MRI, robust, smooth cartilage at second look arthroscopy.**	**100**	**100**	**12 m**	40
Gobbi *et al.*^20^	Prospective study	**8**/53	**MFX**	**Lysholm, Tegner, IKDC and functional tests**	2	1 deep infection, 1 superficial infection	**Improvement in all scores. Better results in traumatic, isolated lesions of MFC. Poorer outcomes in nontraumatic, multifocal lesions.**	/	/	/	66
Gudas *et al.*^21^	RCT	**13**/57	28 OAT vs 29 MFx (all-arthroscopic procedures)	HSS score, ICRS score, ICRS macroscopic evaluation, ICRS repair grade, biopsy, Imaging (MRI & X-ray)	MFX: 9 (at 8.4 m), OAT: 1 (at 3 m).	2 superficial infections in the OAT group	Significant superiority of the OAT over MF for the repair of articular cartilage defects in the knee.	OAT 93%, MFX 52%	/	6.5 m	87
Kacprzak *et al.*^22^	Prospective study	**36**/49	**Scaffold-augmented BMS** **(arthroscopic MFx + Hyalofast scaffold implantation)**	**KOOS, SF-36**	0	0	**Statistically significant improvement in clinical scores. The accelerated rehabilitation protocol shortened the time needed for the athletes to RTS.**	**100%**	**100%**	2.5-3 m	44
Keszég *et al.*^23^	Retrospective study	**55**	**OAT** **(19 arthroscopic, 36 arthrotomy)**	**Tegner, IKDC, Bandi and MOCART 2.0 scores**	/	**Swelling 1, ROM limitation 3, Pain 3, Locking 1. 11 poor Bandi score ratio**	**85% of professional, 34% of competitive, and 88% of recreational players could go back to the same level of sport after surgery.**	**66%**	**91%**	**7.8 ± 3.0**	44
Kon *et al.*^24^	Prospective comparative study	**41**	**20 MFX vs 21MACT** **(arthroscopic Hyalograft C)**	**IKDC, Tegner, EQ-VAS**	**MFX: 0** **MACT: 1 (at 6 y)**	/	**Improvement in all clinical scores in both groups. Similar RTS rate. Faster recovery for MFX but clinical deterioration over time. Longer time to RTS for MACT but more durable clinical results.**	**MFX: 75%**, **MACT: 67%**	**MFX: 80%**, **MACT: 86%**	**MFX: 6.5 m MACT: 10.2 m**	57
Levy *et al.*^25^	Prospective study	**15**	**Arthroscopic debridement**	**MRI, arthroscopic evaluation and second look, Cartilage biopsy (Noyes Classification), Brittberg autologous transplant scoring system.**	**26% new lesions after RTS** **(at mean 1.6 y)**	/	**Debridement allows RTS, but may allow the underlying problem to progress.**	**100%**	**100%**	**10.8 w**	30
Marom *et al.*^26^	Multicenter Descriptive survey	**4,526**	**Nonoperative 50%: PT, IA steroids, IA-HA, IA-PRP, IA-ASC, IA BMAC.** **Operative 50%: debridement, ORIF, BMS, OAT, allograft, ACI/MACT, Coronal osteotomy, TTO**	/	NA	/	**Different management of cartilage lesions worldwide, need for collaborative efforts focusing on establishing consensus guidelines for the optimal management.**	/	**Operative: 75% (IQR: 68-86).** **Non-operative: 80% (IQR: 73-98)**	**Operative: 28w (IQR: 18-30).** **Non-operative: 11w (IQR: 7-15)**	22
Mithoefer *et al.*^27^	Retrospective study	**21**	**MFX**	**Return-to-sport and time continued playing**	/	/	**Restoration of knee joint function in professional football players with high rate of RTS and continued participation.**	**95%**	/	**/**	16
Mithöfer *et al.*^28^	Prospective study	**4**/20	**ACI with periosteal flap (open procedure, “sandwich technique” in 2 pts)**	Lysholm, Tegner, patient evaluation questionnaire for athletic participation and subjective rating of knee function.	/	3 graft hypertrophy	96% good or excellent results with significant increases in postoperative Tegner and Lysholm scores. RTS at same level correlated with shorter preoperative symptoms and a lower number of prior operations.	**100%**	96%	/	44
Mithöfer *et al.*^29^	Prospective study	**45**	**ACI with periosteal flap** **(open procedure, “sandwich technique” if lesion deeper than 1 cm)**	**Tegner, Brittberg**	**6** **(revision ACI)**	**7 patients**	**72% good to excellent results. Successful RTS in significantly younger patient and shorter preoperative duration of symptoms. Combined procedures did not affect RTS.**	**26.7%**	**33%** **(83% in high level, 16% in recreational)**	**18.1 m (14.2 in high level, 22.2 in recreational)**	42
Pánics *et al.*^30^	Prospective study	**61**	**Mosaicplasty**	**HSS Score, Lysholm score, Modified Cincinnati score, ICRS score, Bandi score, Imaging (MRI and x-ray)**	/	**Long-lasting donor site disturbances in 5%**	**Better clinical outcomes in younger patients with smaller lesions. Success rate similar to less athletic patients.**	**67%** **(89% elite and 62% competitive)**	**87%**	**4.5m** **(3.5-6.1)**	46
Zaffagnini *et al.*^31^	Prospective study	**26**/31	**MACT** **(arthroscopic Hyalograft C)**	IKDC, Tegner, EQ-VAS	1 (debridement and meniscal transplant)	0	Improvement in all scores. Previous surgery most influencing factor for RTS at same level.	58.1%	64.5%	/	54
Zarur Mina *et al.*^32^	Retrospective study	**34**	**MFX**	**Modified Cincinnati score, Isokinetic assessment (Cybex) of leg muscles**	**23.5%**	/	**76% excellent—good outcomes. 24% unsatisfactory results, impeding performance to the preinjury level, and consequent retiring from professional football.**	**76.5%**	/	**15.4 w (12-20)**	31
Zmerly *et al.*^33^	Prospective study	**49**	**37 perforations, 12 MFX**	**IKDC, Imaging (X-ray and MRI), second look arthroscopy (only 4 pts)**	**3** **(2 OAT, 1 revision MFX)**	/	**Overall favorable results of perforation for OCD treatment, in cartilage lesions MFX seem to have better results than perforation/drilling**	/	/	/	29

Entries in boldface refer exclusively to data about football players.

pts = patients; MRI = magnetic resonance imaging; y = year; m = months; w = week; RCT = randomized controlled trial; RTS = return-to-sport; ROM = range of motion; IKDC = International Knee Documentation Committee; IQR = Interquartile Range; ICRS = International Cartilage Repair Society; KOOS = Knee Injury and Osteoarthritis Outcome Score; MFX = microfractures; BMS = bone marrow stimulation; OAT = osteochondral autograft transplantation; ACI = autologous chondrocyte implantation; MACT = matrix-assisted autologous chondrocyte transplantation; NA = not assessed.

**Figure 2.**
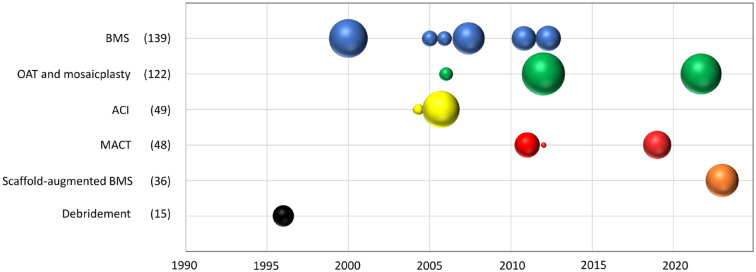
Study publication year and study size according to surgical strategy for cartilage lesions in football players. The figure shows the studies on surgical strategies reported in the literature over time. Each bubble represents a clinical study, and the size of the bubble proportionally represents the number of patients included in the study. The total number of included patients for the different procedures is reported in parentheses. For comparative studies, each group is represented by a bubble. BMS = bone marrow stimulation; OAT = osteochondral autograft transplantation; ACI = autologous chondrocyte implantation; MACT = matrix-assisted autologous chondrocyte transplantation.

Studies were conducted on three continents: North America, Europe, and Asia. Four were in the United States of America,^25,27-29^ four in Italy,^20,24,31,33^ two in Hungary,^23,30^ one in Mexico,^32^ one in Lithuania,^21^ one in Israel,^26^ one in Poland,^22^ and one in Turkey.^19^ Some research teams performed more studies on specific treatment strategies over the years. The trend of publication over time is represented in Figure 2.

The included studies reported the results of 409 football players, 86% men and 14% women, with a mean age of 26.3 years old. All elite, professional, competitive, and recreational athletes were included, and their results were reported at short-term (<24-month follow-up: 24 months in a case report,^19^ mean 19.8 months [range: 14-24 months] in a prospective study on BMS with scaffold augmentation,^22^ and 12 months in a prospective study on debridement)^25^ in three studies, at mid-term (25-96 months of follow-up) in seven studies, and at long-term (>96-month follow-up) in three studies, with two studies not reporting follow-up times. Cartilage lesions had a mean size of 2.9 cm^2^, were located at the medial femoral condyle in 59.7% of the lesions, at the lateral femoral condyle in 24.3%, at the patella in 7.7%, at the trochlea in 6.0%, and at the tibial plateau in 2.3% of the lesions, and were mostly of traumatic etiology (83%). BMS techniques were the most commonly documented (6 studies) in the included studies.^20,21,24,27,32,33^ Other techniques included debridement,^25^ OAT and mosaicplasty,^21,23,30^ ACI,^28,29^ MACT,^19,24,31^ and scaffold-augmented BMS.^22^ Further details of the study populations are reported in Tables 1 and 2, while the available data in terms of outcomes of the different surgical procedures are summarized in Table 3.

**Table 3.** Summary of Patient Characteristics and the Sport-Related Outcomes of the Surgical Strategies.

Procedures	N° patients	Sex M/W	Mean age	Mean lesion size	Rate of RTS	Rate of RTS to the same level	Time to RTS	Failures	Complications
**Debridement**	15 (1 st)	9/6 (1 st)	/	1.1 cm^2^ (1 st, 15 pts)	100% (1 st, 15 pts)	100% (1 st, 15 pts)	2.5 m (1 st, 15 pts)	4 (1 st, 15 pts)	/
**BMS**	139 (6 st)	82/1 (4 st)	25.9 (4 st, 83 pts)	2.8 cm^2^ (2 st, 28 pts)	80% (1 st, 20 pts)	81% (3 st, 75 pts)	4.6 m (2 st, 54 pts)	11 (3 st, 103 pts)	/
**Scaffold-augmented BMS**	36 (1 st)	/	19.8 (1 st, 36 pts)	3.0 cm^2^ (1 st, 36 pts)	100% (1 st, 36 pts)	100% (1 st, 36 pts)	2.5-3 m (1 st, 36 pts)	0 (1 st, 36 pts)	0 (1 st, 36 pts)
**OAT and mosaicplasty**	122 (3 st)	95/21 (2 st)	27.2 (2 st, 116 pts)	2.2 cm^2^ (2 st, 116 pts)	89% (2 st, 116 pts)	67% (2 st, 116 pts)	6.1 m (2 st, 116 pts)	/	22 (2st, 100 pts)
**ACI first generation**	49 (2 st)	32/13 (1 st)	26 (1 st, 45 pts)	5.8 cm^2^ (2 st, 49 pts)	33% (1 st, 45 pts)	33% (2 st, 49 pts)	18.1 m (1 st, 45 pts)	6 (1 st, 45 pts)	7 (1 st, 45 pts)
**ACI third generation (MACT)**	48 (3 st)	48/0 (3 st)	23.9 (2 st, 22 pts)	2.4 cm^2^ (2 st, 22 pts)	87% (2 st, 22 pts)	69% (2 st, 22 pts)	10.3 m (2 st, 22 pts)	1 (2 st, 22 pts)	0 (1 st, 1 pt)

RTS = return-to-sport; st = study; pts = patients; m = months; M = men; W = women; BMS = bone marrow stimulation; OAT = osteochondral autograft transplantation; ACI = autologous chondrocyte implantation; MACT = matrix-assisted autologous chondrocyte transplantation.

In addition, a survey by Marom *et al.*,^26^ conducted over 15 FIFA Medical Centers of Excellence over five continents and including 4,526 football players (10% professional), revealed that half of the cartilage injuries (40% traumatic, mean size 1.5-2 cm^2^) were treated nonoperatively (90% physiotherapy, and 10% different injection therapies). In contrast, the other half underwent surgical treatment through debridement (23%), BMS (40%), and OAT (9%), among other techniques. Nonoperative treatment yielded a return-to-sport rate of 80%, while the rate for operative treatment was 75%. Time to return-to-sport was faster with the nonoperative management versus the surgical approach (11 weeks vs 28 weeks).

### Literature Quality and Risk of Bias

**Figure 3.**
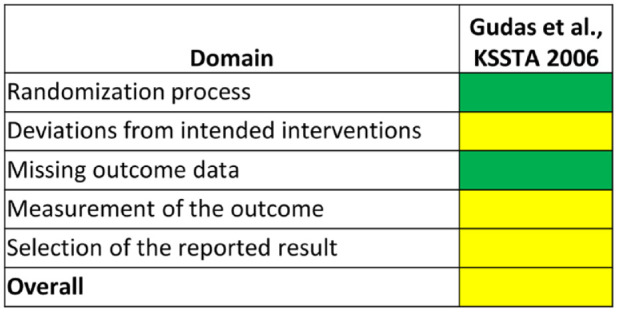
Risk of bias evaluation with RoB 2 tool for RCT. Green: low risk of bias; yellow: some concerns.

The mean modified CMS of the included studies was 43.5 (range: 16-87; Table 2). The RoB 2 was performed for the only RCT included,^21^ reporting overall some concern about the risk of bias (Figure 3).

**Figure 4.**
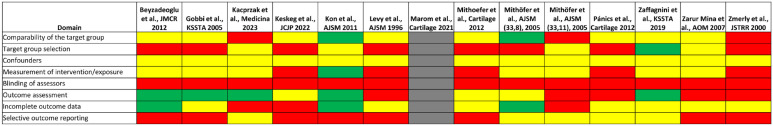
Risk of bias evaluation with RoBANS 2 tool for nonrandomized trial. Green: low risk of bias; yellow: unclear risk of bias; red: high risk of bias; gray: not applicable.

For the other studies, the RoBANS 2 tool was analyzed, reporting an overall high risk of bias. Further details are reported in Figure 4.

### Arthroscopic Debridement

Levy *et al.*^25^ reported a retrospective case series of 15 competitive and professional football players, nine men and six women, affected by isolated full-thickness cartilage injuries with symptoms that included increasing pain, effusion, crepitus, and limited performance. Patients underwent debridement of the cartilage defect to obtain a stable margin of the calcified layer and a bleeding bone at the defect bed with a combination of curette, osteotome, and shaver. Arthroscopic debridement (mean size 112 mm^2^; range 36-225 mm^2^) showed significant pain reduction in all patients, good to excellent outcomes at the Brittberg scoring system at 1-year follow-up, and return-to-sport at an average of 10.8 weeks. However, four patients (26%) developed new lesions after returning to play (average onset 1.6 years after primary surgery).

### BMS Techniques

#### BMS without scaffold

Zmerly *et al.*,^33^ in a case series on 49 football players, observed better IKDC objective outcomes after microfractures than drilling (microfractures: 7 normal, 2 almost normal, 1 abnormal; drilling: 6 normal, 5 almost normal, 6 abnormal, 2 very abnormal) at final follow-up (drilling: 9.7 years; microfractures: 17.4 months). Two patients (4%) with osteochondritis dissecans had to undergo revision procedures with autologous osteochondral transplantation after failed bone-marrow stimulation. One with a cartilage lesion had repeat microfracture in a second-look arthroscopy.

Gobbi *et al.*,^20^ in a prospective study including 53 patients (eight competitive and recreational football players, seven men and one women) with full-thickness cartilage injuries with a mean size of 5 cm^2^ treated with microfractures, found an improvement in the IKDC objective score (one normal, five nearly normal, and two abnormal knees) and Tegner score (mean difference 2.8, from 3.2 to 6) at a mean 72-month follow-up. Eighty percent of patients reported improvement in sport activity during the first 2 years, and gradually decreased to 55% at final follow-up. Outcomes were better in traumatic and isolated injuries of the medial femoral condyle than nontraumatic and multifocal ones. Nevertheless, 30% of the patients had degenerative changes at the imaging evaluation at the final follow-up.

Zarur Mina *et al.*^32^ retrospectively analyzed 34 professional male football players with acute traumatic cartilage Outerbridge III-IV injuries with a maximum size of 6 cm^2^ that underwent microfractures (12 of them with additional arthroscopic partial meniscectomy), finding good to excellent modified Cincinnati scores and a return to preinjury level in 76.5% (26 players) at a mean of 30 months of follow-up. The remaining players had regular or bad results, 11% (4 players) retiring from professional football.

Mithoefer and Steadman^27^ evaluated the return-to-sport rate and continued participation over time in 21 professional male football players with articular cartilage defects after microfractures (two of them undergoing concomitant anterior cruciate ligament reconstruction). Ninety-five percent of the players returned to professional football the next season and played an average of 5 years after the procedure (range: 1-13 years), with no significant reduction in their number of matches per season (from 32 to 28 matches).

#### BMS with scaffold augmentation

Kacprzak *et al.*^22^ analyzed the results of an accelerated full load-bearing rehabilitation protocol after scaffold-augmented BMS implantation for chondral knee lesions in 49 professional athletes, including 36 football players. The surgical procedures included microfractures and the arthroscopic implantation of a hyaluronic-based scaffold. The rehabilitation program, which was the focus of this study, included no brace, full weight-bearing started the day after surgery, supported by crutches for 7 days, without ROM limitation. At a mean 19.8-month follow-up, the authors reported a statistically significant improvement in Knee Injury and Osteoarthritis Outcome Score (KOOS) and SF-36 clinical scores, and all athletes returned to previous sports level in approximately 2.5 to 3 months.

### OAT and Mosaicplasty

Pánics *et al.*,^30^ in a cohort of 61 elite and competitive football players, 55 men and six women, prospectively evaluated clinical and functional outcomes after mosaicplasty of Outerbridge III-IV lesions (mean size 2.4 cm^2^) for mean 9.6 years (range: 2-17 years). They found a significant improvement in the modified HSS, Lysholm, modified Cincinnati score, and International Cartilage Repair Society (ICRS) score. Sixty-seven percent of players returned to the preinjury level (89% of the elite and 62% of the competitive players) at 4.5 months (range: 3.5-6.1 months). Younger patients, patients with smaller lesions, or those located in the femoral condyles benefited the most. The Bandi score revealed that 5% of the patients experienced long-lasting donor site morbidity.

Keszég *et al.*,^23^ a decade later, retrospectively analyzed 55 football players, 40 men and 15 women, with a follow-up of 10 to 25 (mean: 17.5) years after mosaicplasty for 2.0 cm^2^ ± 1.2 lesions (range: 1-5 cm^2^). Twenty-nine percent of the players had undergone previous knee surgeries, including cartilage procedures, and 45.5% had additional procedures during mosaicplasty (16 anterior cruciate ligament reconstructions, seven high tibial osteotomies, and two lateral retinacular releases). Significant improvements were found in the IKDC, and MOCART scores. Eighty-five percent of professional, 34% of competitive, and 88% of recreational players returned to preinjury after surgery at a mean of 7.8 ± 3.0 (range: 4-12) months. The Bandi score revealed that 20% of the patients experienced long-lasting donor site morbidity.

### Autologous Chondrocyte Implantation

#### ACI first generation

Mithoefer *et al.*^28^ evaluated a case series of adolescent athletes that included four football players with osteochondritis dissecans or traumatic cartilage injuries after open ACI with periosteal flap (including autologous bone grafting in two patients). Considering the general study results, most of the adolescents reported significant increases in postoperative Tegner activity scores, Lysholm scores, and returned to high-impact sport. Overall, most patients returned to their previous level or even higher than before. Return to preinjury level was correlated with shorter preoperative symptoms (average 20 months) and fewer prior operations. All adolescents with preoperative symptoms ≤12 months returned to preinjury level, compared to 33% with preoperative intervals longer than 12 months.

Mithoefer *et al.*^29^ also conducted a prospective cohort study following the same surgical technique on football players (33 recreational and 12 professional, 32 men and 13 women) with type IV outerbridge cartilage injuries (size 5.7 ± 0.6 cm^2^) experiencing symptoms for 26 ± 3.4 (range: 3-96) months. Patients had a mean of 2 previous procedures (range: 0-13), and 19 had additional procedures while addressing the cartilage injury (including eight anterior cruciate ligament reconstructions and four tibial tubercle osteotomies). At a mean 41 ± 4 months of follow-up, 72% reported good to excellent outcomes, with a significant overall improvement of Tegner scores. One-third returned to football at a mean of 18.1 months (average of 14.2 months in high level, 22.2 months in recreational). Eighty percent had the same performance level and 87% maintained their ability to play football 52 ± 8 months after surgery. Players who successfully returned to football were significantly younger and had a shorter preoperative duration of symptoms than patients who did not return. Additional procedures in managing cartilage defects did not adversely affect the ability to return to play. Failure was reported in 13% of the patients, including three traumatic graft delaminations and three atraumatic ones.

#### ACI third generation (MACT)

A prospective cohort study by Zaffagnini *et al.*^31^ followed yearly 31 athletes including 26 competitive and professional male football players for 10 years after MACT with a hyaluronan-based scaffold in ICRS grade III to IV traumatic cartilage injuries and osteochondritis dissecans (mean size 2.1 ± 0.7 cm^2^). They found significant IKDC improvement up to the second year, reaching a plateau lasting 10 years. Sixty-five percent of the players were able to return to competition, 58.1% at preinjury level, with activity rates decreasing over the years. Previous surgery (38.7% of the players had undergone ≥1 procedures and 12.9% previous cartilage repair) was the most influencing factor for returning to the previous performance level. One patient (3%) had to undergo arthroscopic debridement, microfractures, and meniscal transplantation 6 years after the primary procedure.

Beyzadeoglu *et al.*^19^ reported a case of a male professional football player with a 9 cm^2^ Outerbridge IV cartilage injury in the lateral femoral condyle undergoing MACT as a revision procedure after two failed microfractures. The player was able to return to his previous level 1 year after the procedure with excellent Tegner–Lysholm and Brittberg–Peterson scores, and tissue resembling healthy cartilage at magnetic resonance imaging (MRI) and second-look arthroscopy.

### Comparative Studies

An RCT on 57 athletes including 13 football players by Gudas *et al.*^21^ compared the outcomes between microfractures and osteochondral transplantation in managing ICRS III to IV cartilage lesions averaging 2.8 ± 0.7 cm^2^ and 2.8 ± 0.7 cm^2^, respectively. At an average of 37.1 months of follow-up, the modified HSS and ICRS scores and functional and objective assessments showed that 96% had excellent or good results after OAT compared with 52% after the microfractures procedure. Athletes following OAT had a higher return-to-sports rate at the preinjury level than those who underwent microfractures (93% vs. 52%) at a mean of 6.5 months (range: 4-8 months) after surgery. Nine patients had to undergo revision procedures after microfractures, eight requiring OAT and one debridement, while only one patient in the OAT group needed a plug substitution. In addition, five OAT and two microfractures had further menisci rupture and one from the microfractures group had ACL injury that required surgical treatment.

In a prospective cohort study, Kon *et al.*^24^ prospectively compared microfractures versus second-generation MACT with a hyaluronan-based scaffold (mean defect size 1.9 ± 0.6 vs. 2.1 ± 0.5 cm^2^, respectively) in 41 professional and semiprofessional male football players. Approximately half of the patients had concomitant ligament or meniscus surgery. Patients in both groups improved all clinical scores (IKDC, EQ-VAS, and Tegner scores) at 2 years. However, IKDC scores were significantly better in the MACT group at a mean final follow-up of 7.5 years. Patients who underwent microfractures showed significant score deterioration over time. Return to competition was similar between microfracture and MACT patients (86% vs. 86%, respectively) but was earlier in the first group (8 months vs. 12.5 months).

## Discussion

The main finding of this systematic review is that different surgical treatments for cartilage lesions yield different results in terms of clinical outcome and return-to-sport rate and time, which are key aspects for the treatment of football players. The current evidence base is limited with large variation in methodological quality and generally a high risk of bias in all studies.

The treatment of cartilage lesions is a rapidly evolving field, with a growing body of knowledge and several strategies proposed to restore the articular surface. While previous attempts to reach a convergence of experts to support decision-making in the daily clinical practice have been partially successful to identify appropriate or inappropriate scenarios in the general population,^34^ currently, there is no consensus for managing cartilage injuries in football players.^35^ This is further complicated by the fact that most football players’ chondral injuries are asymptomatic, which does not warrant an intervention unless they become clinically relevant and impact the athlete’s performance,^36^ and by the management also with less-invasive injective options aiming at a faster recovery and return-to-sport.^26^ Nonetheless, when conservative or injective treatment approaches fail, the surgical solution has to be considered to treat the damaged articular surface and possibly recover joint function for the challenging activities of football players.

The treatment choice may depend on the size of the lesion and the presence of subchondral bone involvement, and age and activity level should also be considered.^37^ The included studies showed a trend for treating cartilage defects smaller than 2.8 cm^2^ using BMS, OAT, and MACT, with a return-to-sport rate ranging from 74.6% to 88%. Among these, microfractures present supporting literature with more data and, excluding the single study about debridement and scaffold-augmented BMS, showed the fastest return-to-sport time (4.6 months), followed by OAT (6.1 months) and MACT (10.3 months). Those results may explain the popularity of microfractures among the FIFA Centers of Excellence around the globe, being the most commonly implemented technique and corresponding to 40% of cartilage procedures.^26^ Surprisingly, the second most widely implemented approach, debridement, was not the subject of any study in professional football players in more than two decades.^25,26,38^

In contrast with the good results reported in the studies included in the present review about football players, there is an increasing body of evidence showing the deterioration of microfractures’ clinical outcomes over time. Professional football players can expect a satisfactory improvement of IKDC and Tegner during the first 2 years, but they could experience an outcome decline at mid-term follow-up, as suggested by different authors.^20,27,39^ Even comparative studies questioned the superiority of microfractures, showing better clinical outcomes after OAT than microfractures (96% vs. 52%), a lower failure rate (4 vs. 31%) and return-to-sports to the preinjury level (93% vs. 52%) in athletes.^21^ Likewise, MACT showed similar outcomes to microfractures in IKDC subjective scores, sports activity levels, rate of return to competition (MACT 86% vs. microfractures 80%), and return to the preinjury level (67% vs. 75%) at 2-years follow-up, but revealed significantly better scores at final follow-up (mean of 7.5 years, minimum 4 years). Microfractures yielded a faster return to competition by a mean of 8 months (vs. 12.5 months), but functional outcomes decreased over time.^24^ More comparative studies are needed to clarify the pros and cons of different surgical techniques. To date, no comparative studies have been conducted comparing OAT versus MACT in professional football players. Still, a retrospective study by Zaffagnini *et al.*^31^ in a general active population showed lower IKDC and Tegner scores for the former in lesions >2 cm^2^, while MACT outcomes were not influenced by the lesion size.

Lesion size seemed to influence the treatment choice in the studies including football players. While debridement was used for the smallest lesions, microfractures, OAT, MACT, and scaffold-augmented BMS were applied for small to mid-size lesions. On the contrary, ACI was used to treat larger lesions with a mean size of 5.8 cm^2^. Although the rate to return-to-sport in an adolescent population of athletes was very encouraging, especially in patients with preoperative symptoms lesser than 12 months, in a population of football players ACI showed a return-to-sports rate of 32.7% at a mean of 18.1 months after surgery.^28,29^ Although these results might be discouraging, the inherent challenges in treating large lesions should be considered, together with the data being related to previous generations of chondrocyte transplantation, which have been subsequently improved both in terms of reduced invasiveness, lowered adverse events, as well as faster recovery with good overall results stable over time.^40,41^ Chondrocyte-based procedures showed to provide a cartilage-like tissue,^42^ which is an advantage with respect to the fibrocartilage obtained with microfractures, and could explain the longer-lasting clinical results documented for MACT compared to microfractures.^39^

The overall results summarized from the available literature showed the advantages of microfractures which supports their use in the clinical practice. At the same time, despite their availability, easy technique, and low cost, microfractures should be used cautiously in football players based on the more multifaceted results derived by a broader assessment of the technique also looking at the stability of the outcome over time. This evidence should be considered when discussing with the injured player and medical team, setting adequate expectations regarding return-to-sports rate, time, and the expected improvement extent, to choose the most suitable approach to address cartilage lesions.

A more recent approach aiming at overcoming the limitations of BMS is represented by scaffold-augmented BMS procedures, entailing the use of a cell-free chondral scaffold as augmentation to microfractures. The overall evidence about this kind of approach is growing,^14^ reporting promising results at short- to mid-term follow-up, and the study by Kacprzak *et al.*,^22^ the most recent found with this review, confirms these results also in professional football players. In fact, the rate and the time to return-to-sport reported analyzing an accelerated rehabilitation program exceed those reported by previous literature, being similar to debridement while including bigger lesions with mean sized 3.0 cm^2^. Nevertheless, this single study at short-term follow-up needs further data in football players and also in the general population the evidence at longer-term is limited.^43,44^ Comparative studies against microfractures confirm the results already shown, with similar results at short-term follow-up^45,46^ but better results at mid-term follow-up,^47,48^ but at this time comparative studies with other techniques are missing.

This study has limitations inherent to the available literature. First, the methodological quality of the included studies is poor and has shown no improvement over the years. Second, some studies presented the results of football players mixed within a broader athlete’s population. Third, the published literature shows a region-based pattern regarding the implemented surgical techniques, which may represent a relevant confounding factor.

Overall, decision-making in cartilage injuries seems to privilege early return-to-sport, making debridement and microfractures the most used techniques. The lesion size influences the treatment choice. However, the current evidence is limited with large variation in methodological quality and a high risk of bias. Further studies are needed to confirm these findings and establish a case-based approach to treat cartilage injuries in football players based on the specific patient and lesion characteristics and the treatments’ potential in terms of both return-to-sport and long-term results.

## Supplemental Material

sj-pdf-1-car-10.1177_19476035231224951 – Supplemental material for Knee Cartilage Injuries in Football Players: Clinical Outcomes and Return to Sport After Surgical Treatment: A Systematic Review of the Literature Supplemental material, sj-pdf-1-car-10.1177_19476035231224951 for Knee Cartilage Injuries in Football Players: Clinical Outcomes and Return to Sport After Surgical Treatment: A Systematic Review of the Literature by Luca Andriolo, Theodorakys Marín Fermín, Giulia Marcella Maryse Chiari Gaggia, Andreas Serner, Elizaveta Kon, Emmanuel Papakostas, Andrew Massey, Peter Verdonk and Giuseppe Filardo in CARTILAGE
